# Case Report: Dual monoclonal antibody therapy in chronic rhinosinusitis with nasal polyps and severe eosinophilic asthma—a proteome analysis

**DOI:** 10.3389/falgy.2024.1484931

**Published:** 2024-11-01

**Authors:** Manon Blauwblomme, Philippe Gevaert, Sharon Van Nevel, Sebastian Riemann, Elke Vandewalle, Gabriele Holtappels, Natalie De Ruyck, Lara Derycke, Anne-Sophie Eeckels, Stijn Vanhee, Bart N. Lambrecht, Guy Brusselle, Thibaut Van Zele

**Affiliations:** ^1^ Department of Otorhinolaryngology, Ghent University Hospital, Ghent, Belgium; ^2^ Upper Airways Research Laboratory, Ghent University, Ghent, Belgium; ^3^ Department of Head and Skin, Ghent University, Ghent, Belgium; ^4^ Department of Respiratory Medicine, Ghent University Hospital, Ghent, Belgium; ^5^ Laboratory of Immunoregulation and Mucosal Immunology, VIB Center for Inflammation Research, Ghent, Belgium; ^6^ Department of Internal Medicine and Pediatrics, Faculty of Medicine and Health Sciences, Ghent University, Ghent, Belgium; ^7^ Department of Pulmonary Medicine, Erasmus Medical Center, Rotterdam, Netherlands

**Keywords:** chronic rhinosinusitis with nasal polyps (CRSwNP), severe eosinophilic asthma, mepolizumab, dupilumab, dual monoclonal antibody therapy, proteomic analysis

## Abstract

**Context:**

Recent insights into type 2 inflammation have led to the development of monoclonal antibody therapies for severe asthma and chronic rhinosinusitis with nasal polyps (CRSwNP). Despite add-on therapy with a monoclonal antibody, some individuals remain uncontrolled in terms of upper and/or lower airway symptoms, prompting an exploration of the efficacy of combining biological therapies and their impact on inflammatory pathways.

**Objectives:**

In this article, we present a distinctive case of a patient with CRSwNP, severe eosinophilic asthma, and uncontrolled upper airway symptoms, who experienced substantial clinical and local inflammatory improvements through dual monoclonal antibody therapy.

**Methods:**

We provide a detailed case description and analysis of the patient's nasal tissue and secretions to gain insights into the local nasal inflammation under this unique therapeutic approach.

**Results:**

The addition of an anti-IL-4Rα antibody led to an improvement in upper airway symptoms and a reduction in both eosinophilic and neutrophilic inflammation, despite prior anti-IL-5 therapy. These effects were consistently observed in both polyp tissue and nasal secretions.

**Conclusion:**

Our patient, with CRSwNP, severe eosinophilic asthma, and uncontrolled upper airway symptoms, experienced substantial improvement with dual monoclonal antibody therapy, without major complications or side effects.

## Introduction

Chronic rhinosinusitis (CRS) has a strong association with asthma, with a prevalence of asthma among CRS patients of about 25%, a sharp contrast to the 5% observed within the general population (1, 2). The classification of CRS includes primary or secondary CRS, further stratified into localized or diffuse disease based on anatomical distribution, with additional categorization into type 2 or non-type 2 disease (1, 3). Approximately 5%–10% of asthmatic patients suffer from severe asthma, which can be divided into type 2-high asthma, characterized by eosinophilic airway inflammation, and type 2-low asthma, defined by neutrophilic and paucigranulocytic features (4). Monoclonal antibody therapies are currently used as part of the medical treatment for a selection of patients with severe asthma and/or chronic rhinosinusitis with nasal polyps (CRSwNP). These monoclonal antibodies target the underlying type 2 inflammation and have shown their efficacy resulting in a significant decrease in asthma exacerbations, nasal polyp size, sinus opacification, severity of symptoms, the need for oral corticosteroids and for sinus surgeries (5–7). Despite advancements, some individuals remain uncontrolled in terms of upper and/or lower airway symptoms.

Our case report offers a unique contribution to the Global Airway Research field as it presents comprehensive clinical findings alongside the collection and analysis of nasal polyp tissue, nasal secretions, and blood samples spanning an 8-year period in a patient undergoing dual monoclonal antibody therapy for CRSwNP and severe eosinophilic asthma. Additionally, a proteome analysis offers valuable insights into the underlying mechanisms associated with this therapeutic approach.

## Case description

In 2014, a 47-year-old patient was referred to pulmonology after an anaphylactic reaction to a nonsteroidal anti-inflammatory drug (NSAID) and 6 months of persistent upper and lower airway complaints. The patient was on medium dose inhaled therapy (ICS/LABA) and Montelukast 10 mg daily, started by the general practitioner. At presentation, he had increased type 2 biomarkers with high blood eosinophilia (1,520/µl or 18.3%), IgE (192.5 kU/L), and FeNO (90 ppb), without systemic manifestations. Lung function test showed an obstructive pattern with a prebronchodilator FEV_1_ value of 2.74 L (68% predicted) and 3.11 L (77% predicted) after bronchodilatation. The patient was diagnosed with a severe, late-onset eosinophilic asthma. Asthma medication was changed to high-dose inhaled therapy (ICS/LABA) with additional low-dose inhaled therapy as needed. Loss of smell and bilateral nasal obstruction led to referral to the ear, nose and throat (ENT) department. Nasal endoscopy showed bilateral small polyps in the middle meatus, with a total nasal polyp score (TNPS) of 2. A diagnosis was made of chronic rhinosinusitis with nasal polyps (CRSwNP), severe eosinophilic asthma, aspirin sensitivity and hypereosinophilia, for which a course of systemic corticosteroids, nasal irrigations with corticosteroids twice daily, and doxycycline 100 mg once daily for 3 weeks, were initiated.

**Figure 1:**
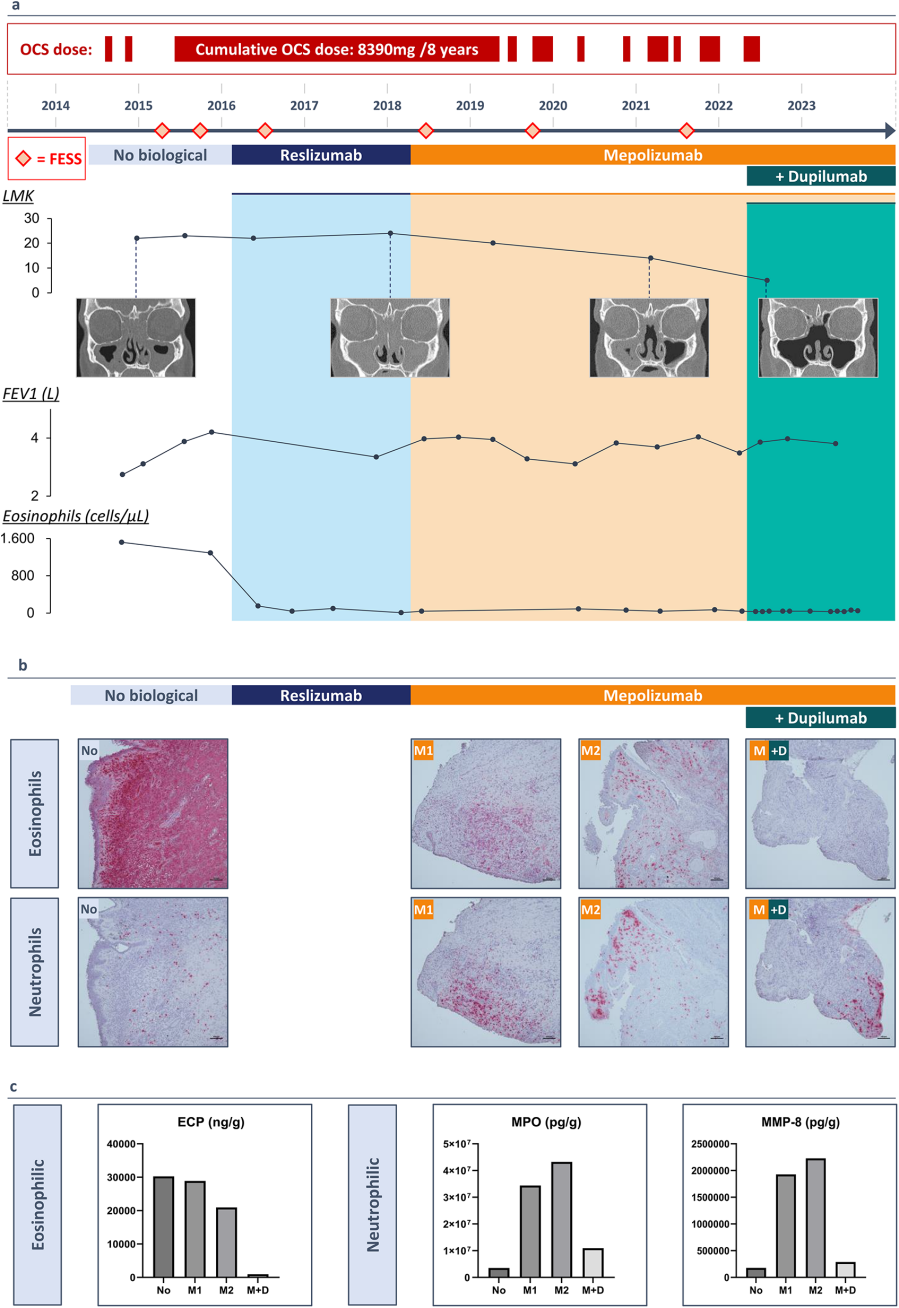
(3 rows, color): **(a)** A timeline of the patients’ medical history (2014-2023) and duration of monoclonal antibody therapy. Lund Mackay Score with resolution of the sinus opacification following add-on therapy with dupilumab, FEV1 values increasing with monoclonal antibody therapy, blood eosinophil count (cells/µl) decreasing with monoclonal antibody therapy. **(b)** Immunohistochemistry staining of polyp tissue for eosinophils with galectin-10 antibody and for neutrophils with elastase antibody. **(c)** Protein measurements of eosinophilic cationic protein (ECP), myeloperoxidase (MPO) and neutrophil collagenase (MMP-8) in nasal polyp tissue before a biological (No), after 20 months of treatment with mepolizumab (M1), after 40 months of treatment with mepolizumab (M2) and after treatment with mepolizumab and add-on treatment with dupilumab (M + D).

Uncontrolled upper airway symptoms necessitated a complete functional endoscopic sinus surgery (FESS) in January 2015. Due to persistent cystic mucus at the level of the frontal sinus, an additional DRAF III procedure was performed. Histological examination of the polyp tissue demonstrated the presence of eosinophils and Charcot Leyden crystals, indicating high eosinophilic activity. By late 2015, high blood eosinophilia (1,290/µl or 12.1%) and uncontrolled symptoms with rapid polyp recurrence persisted. In February 2016, the patient was eligible to participate in a clinical study assessing the efficacy and safety of reslizumab, an anti-IL-5 treatment, given intravenously (3 mg/kg every 4 weeks) (8). Since mepolizumab, another IL-5 targeting agent, became reimbursable in Belgium, the therapy was switched to mepolizumab (100 mg every 4 weeks) after two years, while systemic corticosteroids were continued at a dose of 4 mg every other day. Lung function tests improved, showing a pre-bronchodilator FEV1 of 4.03 L (98% predicted) and 4.07 L (99% predicted) post-bronchodilator after six months of treatment. A good asthma control with mepolizumab and high-dose ICS/LABA was achieved by the end of 2018 and led to the tapering and eventually discontinuation of the systemic corticosteroids. Despite 8 years of treatment and relatively well-controlled asthma, severe upper airway symptoms persisted, requiring 4 additional complete sinus surgeries, antibiotics and a cumulative dose of 8,390 mg of oral corticosteroids (Figure 1a).

**Figure 2:**
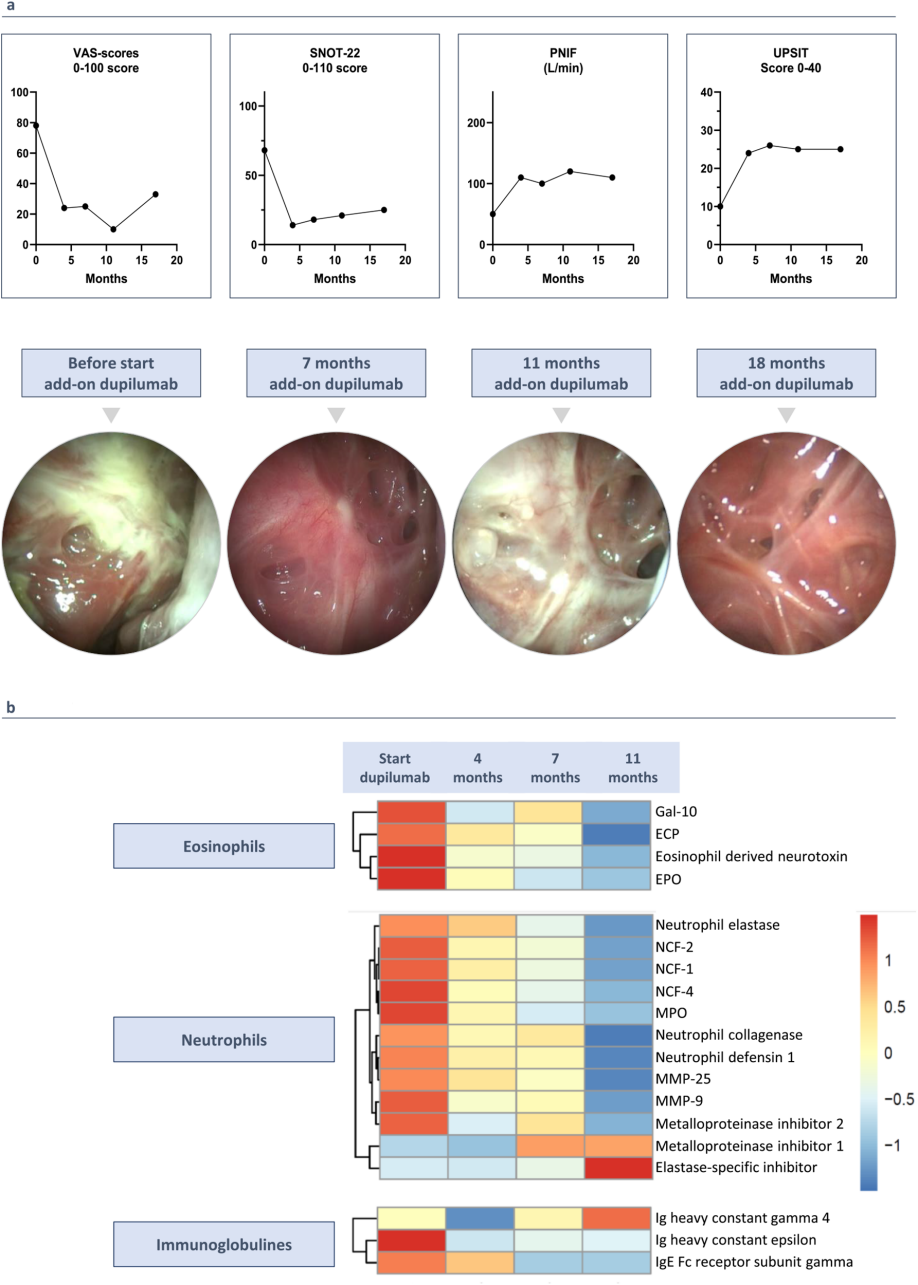
(3 rows, color): clinical outcomes since starting add-on therapy with dupilumab. **(a)** Add-on therapy with dupilumab improves rhinosinusitis VAS-score, SNOT-2 score, UPSIT score and PNIF. Endoscopic images before starting add-on treatment with dupilumab, after 7, 11 and 18 months of add-on therapy with dupilumab. **(b)** Proteome analysis on nasal secretions before and during add-on therapy with dupilumab. Heatmap shows the differentially expressed proteins with LFQ intensity ratios for each protein.

In May 2022, a multidisciplinary team of ENT specialists and pulmonologists decided to add dupilumab, an anti-IL-4Rα antibody, because discontinuing mepolizumab was considered unsafe due to the patient's initially high eosinophil levels and the associated risks of elevated eosinophilia with dupilumab in monotherapy. This decision was driven by the patient's very poorly controlled upper airway symptoms, characterized by persistent purulent secretions and significant symptoms, with a SNOT-22 score of 68 (Figure 2a). The patient started dual therapy (mepolizumab 100 mg every 4 weeks and dupilumab 300 mg every 2 weeks) while continuing high-dose ICS/LABA.

### Clinical outcomes with dual monoclonal antibody therapy

Two weeks after adding dupilumab, the patient experienced relief from upper airway symptoms for the first time in 8 years. After 4 months, significant improvements were observed in the rhinosinusitis VAS-score (symptom severity on a scale of 0–100) and the SNOT-22 questionnaire (scores out of 110), with reductions of 53.0 and 50.0 points, respectively. Peak nasal inspiratory flow (PNIF) increased from 50 to 100 L/min, and the UPSIT smell test showed a 16-point improvement (Figure 2a). Initially, nasal endoscopy revealed very congestive nasal mucosa with purulent secretions, which improved to no congestion and clear secretions (Figure 2a). CT scan showed a 9-point improvement in Lund Mackay score (LMK, score out of 24) (Figure 1a).

Muscle pain, spasms, and elevated blood creatine kinase (CK) levels were observed upon initiation of reslizumab and persisted during mepolizumab treatment. Despite these effects, the patient tolerated the treatment well, and regular monitoring of CK levels and renal function showed results within normal ranges. No adverse events were reported upon the addition of dupilumab. After a year of stable clinical and biochemical values, mepolizumab dosage was tapered. However, after an 8-week interval, the patient's upper and lower airway conditions worsened, leading to the reintroduction of a 4-week dosing interval for mepolizumab, demonstrating the continued necessity for both therapies.

### Eosinophilic and neutrophilic inflammation in polyp tissue during biological treatment

We collected biopsies during each surgical intervention and outpatient visits, providing a unique opportunity to perform immunohistochemical staining for eosinophils with galectin-10 (gal-10) and neutrophils with elastase on polyp tissue samples collected since 2015. Histological examination of the initial surgery revealed the presence of eosinophils and Charcot-Leyden crystals, indicating high eosinophilic activity. Treatment with reslizumab, followed by mepolizumab, reduced eosinophil infiltration in the tissue but resulted in increased local neutrophilic inflammation after 20 months. Upon adding dupilumab, neutrophilic infiltration in the polyp tissue decreased after 18 months (Figure 1b). Protein measurements in the tissue confirmed these observations, showing reduced levels of eosinophilic cationic protein (ECP), which further decreased with dupilumab. Neutrophilic markers, myeloperoxidase (MPO) and neutrophil collagenase (MMP-8), exhibited fluctuations: increasing with reslizumab and mepolizumab, but decreasing with dupilumab (Figure 1c).

### Eosinophilic and neutrophilic inflammation in nasal secretions during biological treatment

The findings of dupilumab on nasal polyp tissue were confirmed by measuring protein levels in nasal secretions before and at 4, 7, and 11 months of add-on therapy. Mass spectrometry analysis identified a total of 3,090 proteins in each nasal secretion sample. The proteome analysis revealed a gradual decrease in neutrophilic markers, eosinophilic markers, high affinity immunoglobulin epsilon receptor (FcƐR1), and Immunoglobulin E, coupled with an increase in Immunoglobulin G4. These down- and upregulations were consistently observed across the four samples. The LFQ (label-free quantitation) intensity ratios for each protein are visually represented in a heatmap, illustrating the dynamic changes induced by dupilumab in the nasal secretions (Figure 2b).

## Discussion

Our patient with CRSwNP, severe eosinophilic asthma, and uncontrolled upper airway symptoms, experienced substantial improvement with dual monoclonal antibody therapy without major complications or side effects. Adding dupilumab improved upper airway symptoms and decreased both eosinophilic and neutrophilic inflammation, despite prior anti-IL-5 therapy. The effects were consistently observed in polyp tissue and nasal secretions. Since dupilumab is known to induce hypereosinophilia when initiated, the treatment with mepolizumab could not be terminated (9, 10). Attempts to reduce mepolizumab led to the recurrence of upper and lower airway symptoms.

The mechanism by which dupilumab reduces neutrophilic inflammation is not well understood. Some studies suggest that IL-4 and IL-13 signaling via IL-4 receptor influences neutrophilic inflammation by affecting neutrophil recruitment, chemotaxis, and function. Blocking this pathway may reduce neutrophilic inflammation (11). However, none of the studies involving anti-IL4Rα specifically examined neutrophil activity before, during, or after treatment. Further research is needed to clarify this.

There is limited research on the efficacy of dual monoclonal antibody therapy in CRSwNP. While some case studies discuss the rationale and safety in asthma, few present clinical outcomes and the effect on local nasal inflammation in CRSwNP (12–14). Non-responders to biologicals among patients with a clear type 2 inflammation highlight the complexity of CRSwNP pathophysiology and the need for further understanding. Research by Van Nevel et al. showed increased concentrations of granulocyte-colony stimulating factor (G-CSF), a cytokine regulating neutrophils, in patients treated with an anti-IgE monoclonal antibody compared to before treatment (15). There is a growing interest in understanding mixed eosinophilic-neutrophilic inflammation and its association to disease outcomes (16, 17). Further research is essential to elucidate underlying mechanisms of treatment resistance and advance personalized CRSwNP management.

Recognizing the limitations of a single case study, such as its uniqueness, underscores the need for further clinical research. Future studies should aim to establish proper patient selection criteria and evaluate the effectiveness of dual monoclonal antibody therapy in CRS patients. Additionally, it is important to investigate the specific types of inflammation both before and during monoclonal antibody treatment in a larger population. A systematic approach to selecting the appropriate drug for specific endotypes is essential, with nasal secretions potentially serving as a non-invasive tool for monitoring endotypes before and during therapy.

In conclusion, this case report highlights the complexities of treating type 2 inflammatory airway disease and demonstrates the benefits of combined monoclonal antibody therapy. The positive outcomes underline the importance of a multidisciplinary approach and the need to target multiple inflammatory pathways.

## Patient perspective

From the patient's perspective, the introduction of dual monoclonal antibody therapy was life-changing. For the first time in 8 years, he experienced significant relief from his upper airway symptoms, which had previously been a constant burden. The return of his sense of smell was particularly exciting, as it reconnected him to simple pleasures he had long missed. The improvements in his breathing and overall quality of life were profound. He is determined to continue the dual therapy, which has restored his quality of life.

## Data Availability

The original contributions presented in the study are included in the article/Supplementary Material, further inquiries can be directed to the corresponding author.
